# Factors associated with receipt of mammogram among caregivers: a comparison with non-caregivers

**DOI:** 10.1186/s12905-020-01079-2

**Published:** 2020-09-29

**Authors:** Soo Young Kim, Yuqi Guo, Chorong Won, Hee Yun Lee

**Affiliations:** 1 Department of Aging and Social Work, College of Nursing, Catholic University of Pusan, Busan, South Korea; 2 School of Social Work, College of Health and Human Services, University of North Carolina at Charlotte, Charlotte, North Carolina USA; 3 School of Data Science, University of North Carolina at Charlotte, Charlotte, North Carolina USA; 4 School of Social Work, University of Alabama, TUSCALOOSA, Alabama USA

**Keywords:** Mammogram, Caregiver, Hours, Non-caregiver, Anderson behavioral model, Cancer beliefs

## Abstract

**Background:**

caregiving responsibilities significantly impact females’ decisions on adhering to preventive mammography. The purpose of this study is to examine (1) the levels of mammogram receipt, (2) the role of caregiving factors on the receipt of mammogram in caregiving group, and (3) the role of cancer beliefs on mammogram screening in caregivers and non-caregivers.

**Methods:**

the 2017 Health Information National Trends Survey (HINTS) provides samples of 1228 women aged 40 to 75 years old for this secondary analysis. By using Andersen’s Behavioral Model of Health Services Use, a binomial logistic regression model was used to analyze associations between mammography and socioeconomic factors, caregiving factors, and cancer belief factors.

**Results:**

caregivers who provided more caregiving hours per week (OR = 0.749, 95% CI = 0.564–0.94) and caregivers who had the belief of rather not knowing the likelihood of getting cancer (OR = 0.673, 95% CI = 0.496–0.914) were less likely to use mammogram. However, caregivers who believed cancer is more common than heart disease (OR = 1.490, 95% CI = 1.302–2.151) were more likely to use a mammogram. Non-caregivers who worried about getting cancer (OR = 1.158, 95% CI = 0.793–1.691) were more likely to use mammogram, but non-caregivers who had the belief of rather not know the likelihood of getting cancer (OR = 0.825, 95% CI = 0.713–0.955) were less likely to use mammogram.

**Conclusions:**

to support caregivers’ breast cancer prevention, caregiving-related policies based on caregiving hours should be developed. Particularly, effort to promote breast cancer screening education and care support among older primary caregivers will likely increase their adherence to preventive mammography uptake. The development of targeted cancer prevention interventions on specific cancer beliefs held by both groups are also urgently needed to promote mammography.

## Background

Breast cancer is the most common cancer and the second leading cause of cancer death among U.S. women [1]. In 2019, an estimated 268,600 new cases of invasive breast cancer and 62,930 new cases of non-invasive breast cancer were diagnosed in women in the U.S.; also, an estimated 41,760 women are expected to die from breast cancer [2]. Previous evidence suggested that the decrease in incidence and mortality rate was partially due to the extensive use of preventive mammograms, which offer early detection and treatment of breast cancer [3, 4]. The latest American Cancer Society breast cancer screening guidelines recommend that “women ages 40 to 44 should have the choice to start annual breast cancer screening with mammograms if they wish to do so; women ages 45-54 should have yearly mammograms; and women ages 55 and older should switch to mammograms every 2 years or can continue yearly screening” [5]. However, getting recommended mammograms is one of the unmet health care among female caregivers [6]. Approximately 23.5% of female caregivers never received a mammogram, particularly [7, 8]. Notably, the difference in mammogram use behaviors between caregivers and non-caregivers is understudied.

Previous studies have reported relevant factors to mammogram use in both caregivers and non-caregiver. For example, age-related trends in mammogram use were observed in both caregivers and non-caregivers in previous studies [3, 9–11]. After 45 years of age, older women were more likely than younger women to have mammograms [9, 12]. Also, cancer beliefs played a critical role in using mammograms. Cancer-related fear was common, which significantly impacted women’s mammograms use [13]. Caregivers tended to be more familiar with cancer than non-caregivers [13–17]. However, non-caregivers seemed to be more attentive to cancer-related self-care and perceive a higher risk of breast cancer than caregivers, which leads to mammograms use [16]. People who have family cancer history, and caregivers of cancer survivors have increased odds in receiving mammograms [18, 19]. In addition, depression is a risk factor for mammography underuse in general populations [20]. Women who are depressed are less likely to receive screening, and female caregivers are at risk of depression due to the heavy caregiver burden [20, 21].

Regarding caregiver-specific factors to mammography screening, previous studies showed mixed or limited results. For example, caregiver burden was identified as one of the barriers to screening [22, 23]. The authors proposed that caregivers who have caregiver procrastination and high burden may lead to less frequent breast examinations [23]; however, another study found no significant association [2]. Also, caregivers of cancer patients generally had an increased likelihood of receiving cancer preventive screenings [23, 24]. An increase of likelihood may be due to the high supply of cancer information from medical professionals, leading to increased awareness of preventive screenings [24]. In addition, financial matter was an aspect impacting mammography recipients [13]. However, no income-related disparities in mammography use have been observed between caregivers and non-caregivers in previous literature.

By using the Andersen’s Behavioral Model of Health Services Use [25], our study compared mammogram screening behaviors between caregivers and non-caregivers to examine (1) the levels of mammogram receipt, (2) the role of caregiving factors, and (3) the role of cancer beliefs on mammogram screening of caregivers and non-caregivers. The hypotheses were:

1. The likelihood of using a mammogram would differently associate with predisposing factors (age, education, and beliefs about cancer) between caregivers and non-caregivers.
2. The likelihood of using a mammogram would differently associate with enabling factors (income, confidence about getting health information, and caregiving burden) between caregivers and non-caregivers.
3. The likelihood of using a mammogram would differently associate with need factors (general health, depression, and family cancer history) between caregivers and non-caregivers.

## Methods

### Research design and data source

This study analyzed data from the 2017 Health Information National Trends Survey (HINTS). HINTS 5’s Cycle 1 (2017) data were collected from January to May, and a single-mode mail survey was generated [26]. According to the latest breast cancer screening guideline that women can start annual screening of mammograms at the age of 40 [5], our study included 1228 women aged 40 to 75 years as samples. The sample was categorized into two subgroups: caregivers and non-caregivers. Overall, the sample consisted of 277 caregiving women and 951 non-caregiving women aged 40 to75 years. More details about the development of HINTS have been reported elsewhere [26].

### Measurement

Caregivers were defined as people who were caring for or making health care decisions for someone with a medical, behavioral, disability, or other condition whether caregiver or not was analyzed as a dichotomous variable (0 = no; 1 = yes).

#### Dependent variable

As National Comprehensive Cancer Network (NCCN) recommended yearly mammography to women ages 40 to 75 and the ACS also recommended women to start yearly mammography at the age of 40 and may continue yearly mammography up to the age of 75, the dependent variable named mammogram screening measured whether a participant had received a mammogram within the past year (12 months). Participants’ self-reported mammogram screening over the past 12 months was analyzed as a dichotomous variable (0 = did not have a recent mammogram screening; 1 = had a recent mammogram screening).

#### Independent variables

Predisposing factors were age (40 to 75), education (1 = Less than 8 years to 7 = Postgraduate), and beliefs about cancer. To assess cancer beliefs, the HINTS included eight items. Six items were assessed by asking respondents to rate on a 4 likert scale (1 = strongly disagree; 2 = somewhat disagree; 3 = somewhat agree; 4 = strongly agree) their cancer beliefs (it seems like everything causes cancer; there’s not much you can do to lower your chances of getting cancer; there are so many different recommendations about preventing cancer, it’s hard to know which ones to follow; cancer is more common than heart disease in adults; when I think about cancer, I automatically think about death; I’d rather not know my chance of getting cancer). Other items (how likely are you to get cancer in your lifetime; how worried are you about getting cancer?) were assessed by asking respondents to rate on a five-point scale (1 = very unlikely; 2 = unlikely; 3 = neither unlikely nor likely; 4 = likely; 5 = very likely, 1 = not at all; 2 = slightly; 3 = somewhat; 4 = moderately; 5 = extremely).

Enabling factors were income (1 = $0–9999 to 9 = ≥$200,000) and confidence about health information (1 = Not confident at all; 2 = A little confident; 3 = Somewhat confident; 4 = Very confident; 5 = Completely confident). We included four additional items that are related to the caregiving characteristic for the caregiver group. The continuous variables included the number of people under their care, and the categorical variables included the caregiving hours per week (1 = < 5 h per week; 2 = 5–14 h per week; 3 = 15–20 h per week, 4 = 21–34 h per week; 5 = 35 or more hours per week), care receiver’s cancer (1 = yes; 0 = no), and care receiver’s chronic illness (1 = yes; 0 = no).

Need factors included four items (general health, depression, ever had cancer, and family ever had cancer). For self-rated health status, participants reported their general health status using a five-point Likert scale (1 = Poor; 2 = Fair; 3 = Good; 4 = Very Good; 5 = Excellent). HINTS contained four items related to depressive symptoms (little interest or pleasure in doing things; feeling down, depressed, or hopeless; feeling nervous, anxious, or on edge; not being able to stop or control worrying). We constructed a depression score by adding a value for the four items that ranged from “not at all” (1) to “nearly every day” (4). We also categorized caregiver’s “Ever had cancer” and “family ever had cancer” to “yes” (1) or “no” (0).

### Data analysis

General characteristics of caregivers and non-caregivers were described by calculating the frequencies, percentages, averages, and standard deviations. We examined the association between independent variables and mammogram screening behavior by conducting a cross-tabulation analysis. Finally, we estimated a binomial logistic regression model that included predisposing, enabling, and need factors as independent variables and dichotomous indicators of mammogram screening behavior as the dependent variable. All analyses incorporated replicated sampling weights provided by HINTS to generate unbiased estimates and were conducted using the Stata 12.0 software package.

## Results

### Characteristics of the sample and rates of mammography

First, Table 1 describes the characteristics of our study sample. Of the 277 in the caregiver group, 176(63.5%) received mammogram screenings. Of the 951 in the non-caregiver group, 601(63.3%) received mammogram screening. Caregivers were younger (56.3 years old, SD = 9.315) than non-caregivers (58.6, SD = 9.222). About 72.4% of the caregiver group had completed some college and higher education, while 33.4% of the non-caregiver group had a high school diploma or less. The majority of both groups reported their health as more than good and not ever having had cancer. The average depression level was higher among the caregiver group (6.291, SD = 3.192) than the non-caregiver group (6.008, SD = 2.959). Two-fifths of participants in both groups reported that their family members have had cancer. More than two-thirds were caring for more than two persons, and most of the caregivers (92.8%) were providing care for less than 20 h per week. Of the caregiver group, 18.8% have provided care for cancer patients, and 38.5% have provided care for patients who have chronic conditions.

**Table 1:** Demographic Characteristics of Caregiver Samples and Non-Caregiver Samples

Variables	Caregiver (*n* = 277)				Non-Caregiver (*n* = 951)			
	Frequency (%)	Screening			Frequency (%)	Screening		
		No (%)	Yes (%)	*x*^2^		No (%)	Yes (%)	*x*^2^
**Dependent Variable**								
Mammogram Screening	176(63.5)				601(63.3)			
**Predisposing factor**								
Age	56.25(9.315)				58.61(9.222)			
Education								
High-school diploma or less	75(27.6)	40.00	60.00	.8494	313(33.4)	41.21	58.79	4.0276*
Some college and higher	197(72.4)	34.01	65.99		623(66.6)	34.51	65.49	
Beliefs about cancer								
Likelihood of getting cancer								
Very unlikely and Unlikely	49(18.5)	38.78	61.22	.8543	180(19.8)	37.78	62.22	0.0439
Neither unlikely nor likely	121(45.7)	32.23	67.77		417(45.8)	36.93	63.07	
Likely and Very likely	95(35.8)	36.84	63.16		314(34.5)	36.94	63.06	
Everything causes cancer								
Agree	186(68.4)	34.95	65.05	.1313	626(67.6)	36.42	63.58	0.0725
Disagree	86(31.6)	37.21	62.79		300(32.4)	37.33	62.67	
Prevention is not possible								
Agree	70(26.2)	38.57	61.43	.2820	245(26.5)	35.10	64.90	0.3740
Disagree	197(73.8)	35.03	64.97		681(73.5)	37.30	62.70	
Too many recommendations								
Agree	202(75.4)	34.16	65.84	.5959	691(74.7)	35.60	64.40	1.5707
Disagree	66(24.6)	39.39	60.61		234(25.3)	40.17	59.83	
Cancer more common								
Agree	117(43.8)	28.21	71.79	4.4746*	421(46.6)	35.39	64.61	.2300
Disagree	150(56.2)	40.67	59.33		482(53.4)	36.93	63.07	
Cancer fatal								
Agree	151(55.5)	35.76	64.24	.0015	524(57.0)	38.36	61.64	1.9121
Disagree	121(44.5)	35.54	64.46		395(43.0)	33.92	66.08	
Rather not know the likelihood								
Agree	89(33.0)	39.33	60.67	.8818	348(37.4)	44.25	55.75	13.5159***
Disagree	181(67.0)	33.52	66.48		583(2.6)			
Worried about cancer								
Not extremely	249(91.9)	37.75	62.25	7.2583**	875(93.9)	37.37	62.63	2.8157
Extremely	22(8.1)	9.09	90.91		57(6.1)	26.32	73.68	
**Enabling Factors**								
Income								
$0–74,999	171(67.1)	37.43	62.57	.0066	557(65.9)	40.57	59.43	5.0811*
≥ $75,000	84(32.9)	36.90	63.10		288(34.1)	32.64	67.36	
Confident about getting health information								
Very confident	155(58.3)	29.68	70.32	5.2092*	561(61.6)	34.76	65.24	1.7880
Not very confident	111(41.7)	43.24	56.76		350(38.4)	39.14	60.86	
Caregiving Characteristic								
Number of people under their care								
One	82(33.7)	39.02	60.98	.8903	–	–	–	–
More than two or more	161(66.3)	32.92	67.08		–	–	–	–
Caregiving hours per week								
< 20 h per week	180(92.8)	33.89	66.11	.4621	–	–	–	–
21–34 h per week	14(7.2)	42,86	57.14		–	–	–	–
Caregiving Cancer (Ref = the others)								
Yes	55(18.8)	34.29	65.71	.0272	–	–	–	–
No	238(81.2)	35.71	64.29		–	–	–	–
Caregiving Chronic (Ref = the others)								
Yes	105(38.5)	39.05	60.95	.9211	–	–	–	–
No	168(61.5)	33.33	66.67		–	–	–	–
**Need factors**								
General Health								
More than Good	221(81.5)	37.10	62.90	1.4774	752(80.3)	34.31	65.69	9.4769**
Less than Fair	50(18.5)	28.00	72.00		185(19.7)	46.49	53.51	
Depression	6.291(3.192)				6.008(2.959)			
Ever had cancer								
Yes	34(12.5)	32.35	67.65	.1713	172(18.3)	29.65	70.35	4.5869*
No	239(87.5)	35.98	64.02		769(81.7)	38.36	61.64	
Family ever had cancer								
Yes	210(78.4)	33.33	66.67	1.2920	696(77.3)	34.20	65.80	5.4602*
No	58(21.6)	41.38	58.62		204(22.7)	43.14	56.86	

Note: * *p* < .05; ^**^p < .01, ^***^p < .001

About 18.5% of the caregiver group thought that they were unlikely or very unlikely to get cancer in their lifetime, and about 68.4% agreed that it seemed like everything could cause cancer. Moreover, nearly 26% of participants reported that there was not much they could do to lower their likelihood of getting cancer, and 75% agreed that there were so many different recommendations about cancer prevention that it was difficult to know which to follow. Nearly half of participants reported that cancer is more common than heart disease (43.8% of caregivers and 46.6% of non-caregivers), and when they think about cancer, they automatically think about death (55.5% of caregivers and 57.0% of non-caregivers). About 33% of caregivers and 37.4% of non-caregivers agreed that they would rather not know their likelihood of getting cancer. Most (91.9% of caregivers and 93.9% of non-caregivers) participants in both groups reported that they were not extremely worried about getting cancer. About 67.1% of the caregiver group and 65.9% of the non-caregiver group members earned <$75,000 per year. About 60% of both groups reported that they felt confident about getting health information.

As can be seen by the cross-tabulated frequencies in Table 1, there were significant relationships between perceiving cancer as more common than heart disease (χ 2 = 4.4746, *p* < 0.05), worries about cancer (χ 2 = 7.2583, *p* < 0.01), confidence about getting health information (χ 2 = 5.2092, *p* < 0.05), and getting mammogram screenings in the caregiver group. Moreover, in Table 1, there were significant relationships between education (χ 2 = 4.0276, *p* < 0.05), rather not know the likelihood (χ 2 = 13.5159, *p* < 0.001), income (χ 2 = 5.0811, *p* < 0.05), general health (χ 2 = 9.4769, *p* < 0.01), ever had cancer (χ 2 = 4.5869, *p* < 0.05), family ever had cancer (χ 2 = 5.4602, *p* < 0.05), and taking mammogram screenings in the non-caregiver group.

## Multivariate analysis

### Binominal logistic regression

Estimates from the binominal logistic regression model presented in Table 2 show that mammogram screening was positively associated with age (OR = 1.058, 95% CI = 1.022–1.095, OR = 1.029, 95% CI = 1.013–1.046) and negatively with “rather not know my likelihood of getting cancer” (OR = .673, 95% CI = 0.496–0.914, OR = .825, 95% CI = 0.713–0.955) for both groups. However, among the caregiving group, the dependent variable was positively associated with confidence in getting health information (OR = 1.432, 95% CI = 1.049–1.955) and “cancer is more common than heart disease” (OR = 1.490, 95% CI = 1.032–2.151) and negatively associated with caregiving hours per week (OR = .749, 95% CI = 0.564–0.994). For the non-caregiver group, the dependent variable was positively associated with how worried they were about getting cancer (OR = 1.156, 95% CI = 1.000–1.337) and negatively associated with depression (OR = .919, 95% CI = 0.871–0.969).

**Table 2:** Logistic Regression on Receipt of Mammogram Screening by Caregiving and Non-Caregiving Group

Factors	Predictors		Caregiver		Non-Caregiver	
			OR	95% CI	OR	95%CI
**Predisposing factors**	Age		1.058^***^	1.022, 1.095	1.029^***^	1.013, 1.046
	Education		1.096	0.887, 1.355	1.093	0.980, 1.218
	Beliefs about cancer	Likelihood of getting cancer	1.019	0.895, 1.160	.932	0.866, 1.003
		Everything causes cancer	.971	0.681, 1.384	1.183	0.983, 1.423
		Prevention is not possible	.777	0.550, 1.099	.989	0.823, 1.188
		Too many recommendations	1.158	0.793, 1.691	1.060	0.876, 1.282
		Cancer more common	1.490^*^	1.032, 2.151	1.126	0.939, 1.349
		Cancer fatal	1.200	0.854, 1.685	.916	0.773, 1.085
		Rather not know the likelihood	.673^*^	0.496, 0.914	.825^**^	0.713, 0.955
		Worried about cancer	1.213	0.916, 1.606	1.156^*^	1.000, 1.337
**Enabling Factors**	Income		1.074	0.927, 1.243	1.035	0.962, 1.113
	Confident about getting health information		1.432^*^	1.049, 1.955	1.021	0.868, 1.201
	Number of people under their care		1.523	0.889, 2.609	–	–
	Caregiving Hours per week		.749^*^	0.564, 0.994	–	–
	Caregiving Cancer (ref = others)		.735	0.306, 1.769	–	–
	Caregiving Chronic (ref = others)		.657	0.370, 1.166	–	–
**Need factors**	General Health		.803	0.571, 1.128	1.138	0.952, 1.359
	Depression		0.937	0.849, 1.034	.919^**^	0.871, 0.969
	Ever had cancer		0.696	0.281, 1.723	1.351	0.899, 2.030
	Family ever had cancer		1.404	0.695, 2.837	1.344	0.956, 1.891
**Number of observations**			277		951	
**Pseudo R**^**2**^			0.124		0.057	
**Log Likelihood Rate Test**			43.05		67.51	

Note: ORs Odds ratios, * *p* < .05; ^**^p < .01, ^***^p < .001

## Discussion

Our study results revealed similar mammogram screening rates for caregivers and non-caregivers(63.5% vs. 63.3%). Women caregivers within our sample did not neglect their breast cancer screening needs, which is consistent with the previous findings [17]. The findings of this study partially support our hypotheses. Age was identified as a positive factor for both groups, which is a promising finding in light of the importance of screening older women who are at increased risk for breast cancer. Previous studies have well documented that the risk of breast cancer increases with age [27]. The risk of having breast cancer increases after 40 years old and most breast cancers are diagnosed among older women who are 50 and older [27, 28].

Regarding predisposing factors, caregivers and non-caregivers identified different cancer belief factors associated with the utilization of mammograms. Among non-caregivers, the worry of getting cancer was a significant predictor of using mammograms. A recent study reported that women who worry about getting breast cancer were more willing to adhere to mammograms [29]. However, our study suggested that this knowledge could not be applied to caregiver populations.

In turn, caregivers identified the belief that cancer is more common than heart disease as a significant predictor in the utilization of mammograms. Also, access to health-related knowledge was positively associated with mammogram use among caregivers. The heightened level of health-related knowledge due to caregiving experience and easier access to medical professionals may help caregivers receive mammograms [23].

In addition, unwilling to know their possibility of getting cancer was a significant predictor of using mammograms for both caregivers and non-caregivers. The majority of the respondents did not want to know their likelihood of getting cancer and associated cancer-related death. These findings add to evidence that fear of having cancer is a significant predictor of not receiving a mammogram, which is supported by a previous study [30].

For enabling factors, mammogram screening behavior was negatively associated with hours of caregiving among caregivers. Even though there was not a significant difference in mammography rates between caregivers and non-caregivers, disparities in mammogram use exist within caregivers’ groups. Caregivers who have more caregiving hours per week were significantly less likely to use mammograms. One previous study found that female caregivers who provide more than 14 h per week of caregiving had significantly lower odds of receiving mammography [8]. The overwhelming caregiving hours led to the underuse of mammograms as they were not able to take time off to care for themselves [8].

Finally, regarding need factors, non-caregivers, who showed symptoms of depression, exhibited lower odds of having mammograms. Depression is a risk factor for the underuse of mammography because depression generally leads to self-care neglect, including using mammograms [31, 32]. In this analysis, no other need factor associated with mammogram use among caregivers at a significant level.

### Limitation

Our study had several limitations. First, as a secondary analysis, we were unable to examine the impact of details regarding the caregiving situation on mammogram screening behaviors. Even though the HINTS provided essential information on caregiving status, the information on caregiving duration and situation is lacked, such as hours of caregiving, the reason for caregiving, and relationship to the care recipients. However, our study is also strengthened by the high quality of the HINTS, its sampling procedures, and nationally representative samples. Second, the effects of caregiving by race were unable to be examined. Racial disparities in mammography have been well documented for both caregivers and general women [33, 34]. Our study focused on comparing mammogram screening behaviors between caregivers and non-caregivers. Third, our study was unable to compare mammogram use between women caregivers and non-caregivers with a 24-month time frame for repeated screening, given the possible differences in advice among women ages 40 to 44 and women ages 55 years older receive.

## Conclusion

Our results demonstrate that there was no difference in receipt rates of mammograms between caregivers and non-caregivers. However, when considering caregiving status, women who need to spend more hours on caregiving may neglect their breast cancer screening needs. Policymakers should consider providing free respite care service or preventive-care-related time off work to facilitate caregivers’ early detection of breast cancer. Also, the effects of different cancer beliefs (e.g. rather not know the screening results; worry about cancers) on mammography behaviors varied between caregivers and non-caregivers. Education programs on breast cancer care and mammograms need to be designed with considering participants’ caregiver status. For caregivers, education should focus on the importance of early detection of cancer survival. For non-caregivers, education should focus on reducing the fear of cancer. Future studies may analyze the caregiving roles on repeated mammograms (yearly or bi-yearly) with age-stratified samples.

## Data Availability

Health Information National Trends Survey is a public use dataset that is available from https://hints.cancer.gov/data/default.aspx.

## Abbreviations

HINTS: Health Information National Trends Survey
